# Swine to human transmission of reassortants of pandemic (H1N1) 2009 and endemic swine influenza viruses: Abstract

**DOI:** 10.1371/currents.RRN1285

**Published:** 2011-11-16

**Authors:** U. Chandimal de Silva, Teruo Yasunaga

**Affiliations:** ^*^Scientist, Immunology Frontier Research Center, Osaka University. and ^†^Osaka University

## Abstract

To gain insight into the possible origin of a new reassortant influenza A virus between pandemic (H1N1) 2009 and endemic swine viruses that has jumped the species barrier and caused a few infections among humans in Indiana and Pennsylvania recently, we analyzed all full genome sequences related to this virus and report its evolutionary history, but failed to determine how the virus had emerged simultaneously in two geographically distinct areas.

## **Introduction**

On 2 September, 2011, the Centers for Disease Control and Prevention(CDC) in the United States reported two cases of febrile respiratory illness in two infants[1] in Indiana (patient A) and Pennsylvania (patient B), both caused by a swine-origin influenza A (H3N2) virus with a matrix(M) gene acquired from the pandemic (H1N1) 2009 virus. One of the patients (patient B) has had direct exposure to swine at an agricultural fair in Washington County, Pennsylvania, four days before she developed symptoms, while no such exposure was identified for patient A. On 5 September, the Pennsylvania Department of Health reported two additional cases of human infection in Pennsylvania among two girls below 10 years, both of whom incidentally have attended the same agricultural fair[2] [3].

Human infection of swine origin influenza viruses is not uncommon[4]. However, given the geographical distance of more than 400 kilometres between Washington County and the border of the State of Indiana(http://g.co/maps/xgqxd) and the simultaneity of the incidents, coupled with the novelty of the viral genotype, we decided it was important to fully analyze the genetic sequences in order to gain a better understanding of the evolutionary origin and epidemiology of this new reassortant strain.         

## **Methods and results**

The full genome sequences of A/Indiana/08/2011 (patient A), A/Pennsylvania/09/2011 (patient B), and A/Pennsylvania/11/2011, and all available segments - HA, M and NS - of A/Pennsylvania/10/2011 were downloaded from GenBank/EMBL/DDBJ (Accession numbers JN655532-JN655558) and carefully studied for similarities after performing multiple alignment. All available sequence data for the latter two strains were identical, hence A/Pennsylvania/10/2011 was left out of further analysis.

The genomes sequences of PA/11/11 and IN/08/11 were virtually identical (nine nucleotide differences; three of them non-synonymous) , supporting the case for a human host lead inter-state transportation, because any other scenario would place a minimum of two cross species transmission events between these two isolates with almost identical genome sequences. PA/09/11, on the other hand, had drifted away slightly from the rest of the strains, with unique nucleotides at 21 positions along the genome. Nevertheless, most of these (18/21) represented synonymous substitutions and the gene composition of all the reassorted viruses were the same, making it reasonable to assume that they were derived from a common ancestor which was the product of a distant reassortment event in swine.

We also confirmed the fact that the matrix gene of the new cluster was derived from the pandemic (H1N1) 2009 lineage, while all other gene segments were essentially derived from the now widely circulating triple reassortant swine H3N2 viruses(trH3N2)[5] [6], except for PB1 which was more closely related to A/Swine/Kansas/77778/07, a highly virulent reassortant H1N1 virus[7].

We further analysed these genome sequences with nine reference genomes with known sampling dates chosen from among the closest human and swine sequences for each segment, in order to infer their phylogenies. The Hasegawa-Kishino-Yano model with gamma-distributed rates among sites (HKY+Γ) was used within a relaxed molecular clock framework where evolution rates were allowed to vary among lineages. Phylogenies for each segment were sampled from a Bayesian Markov chain Monte Carlo(MCMC) simulation[8] of at least 50 million steps, after confirming convergence and removing the first 10% as "burn-in".

The phylogeny for the matrix gene clearly shows the pandemic (H1N1) 2009 origin of the new cluster, while the other phylogenies support the trH3N2 origin of the other gene segments (Figure). Moreover, the closer phylogenetic relationship between the new cluster and A/Swine/Kansas/77778/07 in PB1, suggests other reassortment events among swine viruses may have played a role in its evolution.

**Figure:**
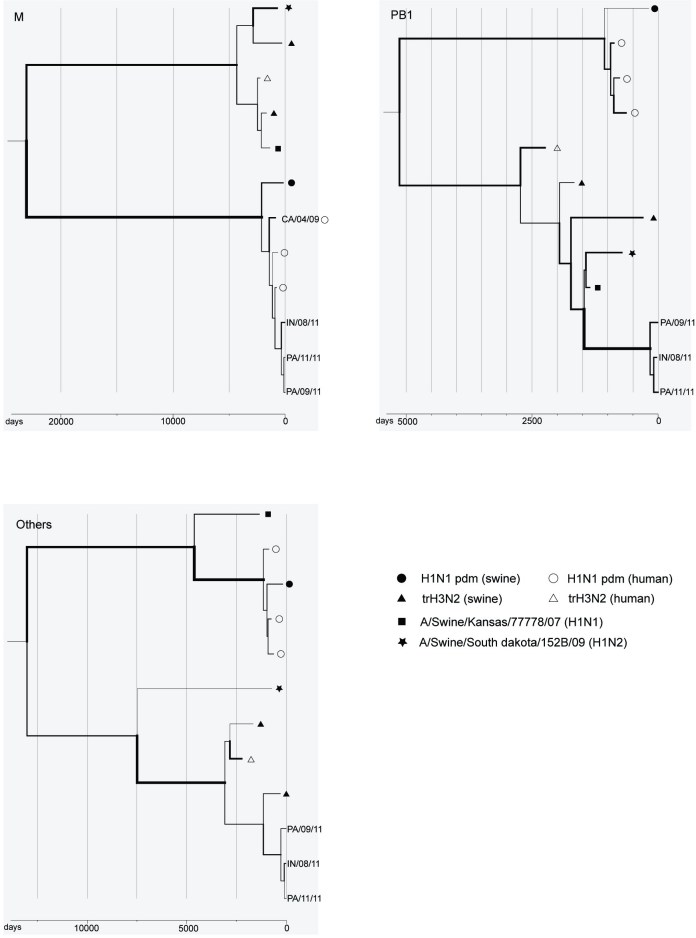

**Figure.  Phylogenetic relationship between the new reassortant cluster and its parent strains for M, PB1, and all other gene segments concatenated together.**

The x-axes denote the sampling dates back numbered from 1 September 2011. Different scales are used for the three trees due to vast differences in divergence times. The line width is proportional to the estimated rate of evolution along different branches. The reference strains for H1N1 pdm are A/California/04/09, A/Australia/32/09, A/Boston/701/09 and A/Swine/Ohio/9838/11, and those for trH3N2 are A/Ontario/RV1273/05, A/Swine/Minnesota/1300/07 and A/Swine/Pennsylvania/62170-1/10.    

## **Discussion**

A recent publication describing several multiple reassortant influenza A viruses isolated in the north-eastern United States during routine surveillance of pigs[9] gives valuable insight into the epidemiology of swine influenza in the region. All nine viruses reported had multiple genes derived from pandemic (H1N1) 2009 virus, whereas most other genes showed high (>97%) nucleotide identity with trH3N2 viruses A/Ontario/RV1273/05 and A/Swine/Minnesota/1300/07. One striking characteristic is that the matrix gene of all viruses were derived from pandemic (H1N1) 2009, and it is the only segment that had the same gene constellation across all nine reassorted viral genomes with various unique reassortment patterns. This is not so surprising though, given other recent findings[10] from experiments in guinea pigs on the critical role of the M gene in pandemic (H1N1) 2009 viral transmission.

The recent reassortant cluster is very similar in its high (>97%) nucleotide identity with the trH3N2 viruses in its internal genes except M, as well as in the common origin of its M gene. While we now know that multiple reassortant pandemic H1N1/swine trH3N2 viruses have been circulating well in swine lately, the first such virus detected in humans may well be the one that has best fitness for transmission in humans. In other words, the only gene from the 2009 pandemic virus that was 'retained' in the human virus (M gene), which also happens to be one of the two genes introduced into the precursor of the 2009 pandemic virus just before it jumped the species barrier[11], may well be the necessary and sufficient addition to trH3N2 required for sustained transmission in humans. The importance of increased surveillance in both hosts and double-checking of 'unsubtypable' infections in humans at this time may not be overemphasized.  

### **Acknowledgements**

We are grateful for assistance provided by Dr. Scott Epperson of Influenza Division, CDC, in locating the viral nucleotide sequences used in our analysis, and thankful for helpful comments on a previous version of the manuscript from an anonymous reviewer.

### **Funding Information**

We would like to acknowledge the support from Osaka University for this study. 

### **Competing Interests**

The authors have declared that no competing interests exist.
